# Exploring the Role of Social Support between Discharge Teaching and Readiness for Discharge in Ocular Fundus Disease Patients: A Cross-Sectional Study

**DOI:** 10.1155/2021/5547351

**Published:** 2021-06-17

**Authors:** Jie Zhang, Shuyu Yao, Feifei Huang, Yan Zhang, Nanqi Huang, Huiming Xiao, Jingping Zhang, Yu Lian

**Affiliations:** ^1^Nursing Psychology Research Center, Xiangya School of Nursing, Central South University, Changsha 410013, China; ^2^State Key Laboratory of Ophthalmology, Zhongshan Ophthalmic Center, Sun Yat-sen University, Guangzhou 510060, China; ^3^Nursing School, Fujian Medical University, Fuzhou 350122, China; ^4^Department of Nursing, Jiangxi Health Vocational College, Nanchang 330052, China; ^5^Department of Gastroenterology, The Third Affiliated Hospital of Guangzhou Medical University, Guangzhou 510150, China

## Abstract

**Background:**

This study aims to evaluate the quality of discharge teaching and readiness for discharge of fundus disease patients treated with day surgery and understand the role of social support between them.

**Methods:**

This was a cross-sectional descriptive correlational survey. Through convenient sampling, fundus disease patients treated with day surgery from Zhongshan Ophthalmic Center, China, were recruited. Data were collected using demographic and disease-related information, quality of discharge teaching scale, readiness for hospital discharge scale, and social support scale.

**Results:**

255 fundus disease patients treated with day surgery were recruited at last. The mean total score of readiness for discharge, quality of discharge teaching, and social support in patients with fundus disease were 157.91 (SD = 26.68), 122.97 (SD = 21.55), and 36.32 (SD = 7.60), respectively. Participants with stronger social support had better discharge teaching and then had higher readiness for discharge. Social support played a partial mediator role in the relationship between discharge teaching and readiness for discharge. The mediation effect ratio was 5.5%.

**Conclusions:**

The quality of discharge teaching and social support among fundus disease patients who underwent day surgery was relatively high, and readiness for discharge was good. Social support is essential for the quality of discharge teaching and the improvement of discharge readiness. Clinical nurses need to provide appropriate guidelines to help patients seek effective support and improve quality of discharge teaching to enhance the readiness for discharge of fundus disease patients treated with day surgery.

## 1. Introduction

Ocular fundus disease refers to various retinopathy and optic neuropathy, which includes the pathological changes of vitreous, retina, choroid, and optic nerve, such as inflammation, tumor, vascular diseases, various degenerative diseases, and many systemic diseases caused by eye diseases [1]. Ocular fundus disease is an important cause of blindness in China today. It has been a serious threat to people's health and quality of life [2]. Antivascular endothelial growth factor (VEGF) medication, photodynamic therapy (PDT), minimally invasive vitrectomy, and intraocular injection are the main surgical treatment methods for this disorder [3]. In recent years, as an emerging medical model, day surgery is more and more widely popular [4]. Day surgery refers to patients' admission, operation, and discharge within 24 hours, and it has the advantages of increasing bed occupancy rate, decreasing economic burden of patients, and educing doctor-patient conflicts [5]. Fundus disease surgery is one of the day surgical modes of fundus disease, which has the advantages of short operation time, fast recovery, good general condition of patients, and low risk of anesthesia (mainly local anesthesia) [6]. After a period of observation in the day ward, patients who underwent day surgery return home to self or family care. Whether the day surgery patients experience postoperative complications and have good rehabilitation depends on adequate care of their family members and themselves [7]. Discharge preparation services in the day ward are therefore extremely important.

Readiness for hospital discharge has been described as an estimate of patients' and family members' ability to leave an acute care facility [8], a perception of being prepared or not prepared for hospital discharge [9, 10], and as an indicator of sufficient recovery to allow safe discharge although the patient is in an intermediate rather than later stage of recovery [11]. Evaluation of readiness for hospital discharge may prevent patients from being discharged prematurely, reduce the incidence of complications and readmission rates after discharge, can also save medical costs, and reduce the burden on medical resources [12]. Several research studies focused on the readiness for discharge among postpartum mothers who experienced an uneventful vaginal or cesarean birth of a healthy infant [13], adult patients in general ward [14], and patients who have surgery [15, 16]. The readiness for discharge of patients with ocular fundus disease after day surgery has not been assessed.

Previous studies have shown that readiness for discharge had a positive association with discharge teaching [17–20]. As one of the primary strategies and a basic part of discharge planning services, discharge teaching can facilitate the patients' readiness for discharge to go home in a transition period and improve the cure rate and self-management outcomes, including preventing complications [18, 21], lowering emergency room visits, rehospitalizations, and symptoms [22]. When nurses, doctors, and other medical staff give patients and their family members information of medical care in the form of education or communication [23], the barrier that commonly comes up was that the content of education may be delivered in a rushed manner, without individualization of the content based on a patients' needs [18]. Thus, individual education methods used in inpatient wards are not suitable in day wards [7]. Presently, little is known about how discharge teaching affects discharge readiness, especially for Chinese patients with ocular fundus disease following day surgery.

Social support refers to the various types of free assistance from a social network, which may be formal and/or informal, including emotional and physical support [24]. The research demonstrated that social support is one of the most critical and effective factors in helping adolescents and adults cope with and adjust to life changes [25], such as hospital discharge. In addition to discharge teaching, social support might also have an association with readiness for hospital discharge [26–28]. That is, social support can increase patients' readiness for discharge for some patients in hospital through having appropriate support from knowledgeable healthcare professionals [29, 30]. Besides, friends, family, and supporters of patients may provide information and tangible forms of support; then patients can feel empowered when in a supportive social environment, which in turn could encourage them to engage in recovery-promoting behaviors [31]. When fundus disease day surgery patients are discharged, they are still in the recovery phase instead of being fully recovered, and their physical and mental conditions, disease knowledge, and skills may be insufficient, leading to their self-management and self-care which are very important to the recovery of eyesight after discharge, mainly borne by the patients themselves or their family. The effectiveness and quality of discharge teaching for patients also depend on the patient's social support. Thus, social support probably has been displayed as an important factor for discharge teaching and readiness for hospital discharge of patients based on the literature review. However, an explanation of the role of social support in the relationship between discharge teaching and readiness for hospital discharge in Chinese patients with ocular fundus disease treated with day surgery is lacking.

**Figure 1:**
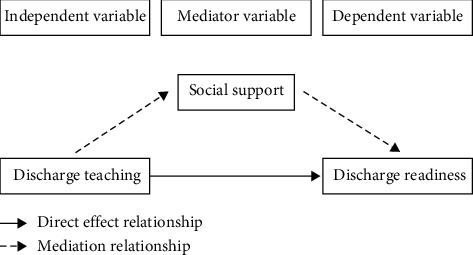
Hypothetical relationship diagram.

In order to obtain useful information for nurses or medical staff in preparing a better discharge teaching and outcomes, it is essential to assess the quality of discharge teaching, social support, and readiness for hospital discharge among patients with ocular fundus disease treated with day surgery. Based on previous literature reviews, we hypothesized that social support may mediate between discharge teaching and readiness for discharge (Figure 1). The objective of study was to guide the role of social support in the relationship between discharge teaching and readiness for discharge, which can promote the development of targeted interventions.

## 2. Materials and Methods

### 2.1. Study Design and Sample

The study was a cross-sectional descriptive correlational survey. The researcher recruited patients with ocular fundus disease from China through convenient sampling from October 2017 to June 2018 (this study was executed and reported in accordance with STROBE Statement). The inclusion criteria were patients (1) ≥18 years old; (2) diagnosed with ocular fundus disease and underwent surgery in the day ward; (3) stable after surgery; and (4) able to communicate in Chinese and willing to participate in the study and gave signed informed consent. The exclusion criteria were (1) having severe cognitive impairment and hearing impairment; (2) having other serious life-threatening diseases; and (3) participating in other relevant researches.

Kendall's sample size calculation principle yields sample sizes 5–10 times the number of variables [32]. In our research, there were 19 variables (7 related to social demographic information, 2 to disease-related information, 3 to the quality of discharge teaching, 3 to social support, and 4 to readiness for hospital discharge). Therefore, the sample size in this study was set from 114 to 228 (19 × 5 × 1.2 = 114–19 × 10 × 1.2 = 228).

### 2.2. Ethical Considerations

The study was approved by the institutional review board of our hospital, and all eligible participants signed the informed consent before they completed the questionnaire. They were informed of their rights and can withdraw the research at any time.

### 2.3. Measurement

#### 2.3.1. The General Information Questionnaire

The characteristics of patients with ocular fundus disease were collected by the self-compiled demographic questionnaire, including age, gender, marital status, educational level, monthly income, living place, insurance, ophthalmology history, and ophthalmic surgery history.

#### 2.3.2. The Quality of Discharge Teaching Scale (QDTS)

The quality of discharge teaching scale (QDTS) was used to measure the perceptions of surgical patients regarding the quality of discharge teaching and it was developed by Weiss and Piacentine [12]. The Chinese version of QDTS was revised by Wang et al. [33] and authorized by Weiss. The Chinese version of QDTS consists of 18 items classified into three dimensions, included teaching contents that patients thought they needed (6 items), teaching contents that patients obtained (6 items), and teaching skills and effectiveness (12 items). The item used a scoring method of 0 to 10 points. The total scores ranged from 0 to 240, and a higher total score indicated better quality of discharge teaching [12]. The scale has good reliability and validity and can effectively measure the quality of discharge teaching among patients [18, 34]. The reliability coefficient of the Chinese version of QDTS among Chinese patients was 0.924 [33]. The reliability of the scale in this study was 0.865.

#### 2.3.3. Social Support Rating Scale (SSRS)

The scale was developed by Xiao [35]. The SSRS consists of three dimensions including subjective support, objective support, and support utilization. A higher total score indicated higher levels of social support. The scale has good reliability and validity and it was considered as a qualified tool for assessing social support of Chinese [36]. The reliability of the scale in this study was 0.805.

#### 2.3.4. Readiness for Hospital Discharge Scale (RHDS)

This scale was used to measure the readiness of hospital discharge for patients. It was developed by Weiss et al. [17]. The Chinese version of the RHDS was revised by Zhao et al. [37] and authorized by Weiss. The Chinese version of RHDS consists of four dimensions: physical conditions, mastery of disease knowledge, coping ability after discharge, and available social support. Higher scores reflect higher levels of readiness of hospital discharge. The scale has good reliability and validity [20]. The reliability of the scale in this study was 0.916.

### 2.4. Data Collection

Data were collected from Zhongshan Ophthalmic Center in China by convenient sampling from October 2017 to June 2018. Generally, nurses carried out postoperative education within half an hour after the operation of patients. The content of the education mainly includes posture guidance, vital signs measurement, diet guidance, eye hygiene, and eye protection. And nurses provided discharge teaching 2 hours before patients were discharged and reemphasized the content of postoperative guidance, such as taking transportation, activities, and rest in life and how to take medicine and return to hospital for review. The researcher collects the questionnaire half an hour before patients discharge. The data collection was completed by a researcher and a research assistant. The researchers explained the purpose and significance of the study to participants; then, they introduced the contents of the questionnaire to participants and explained how to complete the questionnaire. Questionnaires were collected by the researchers directly after completion.

### 2.5. Statistical Analysis

Statistical analyses were conducted with IBM SPSS Statistics version 20.0 (SPSS Inc., Chicago, IL, USA). Descriptive data were presented as means and standard deviations. Pearson's correlation analyses were used for correlations among discharge teaching, social support, and readiness for hospital discharge. Analyses of variance and independent sample *t*-tests were used to analyze the significance of sociodemographic differences among variable scores.

According to the methods used in many studies [38, 39], 3-step analysis was used to test the mediating effects. Firstly, regression analysis was performed using discharge teaching as the independent variable and social support as the dependent variable. Secondly, readiness for hospital discharge as the dependent variable was regressed on discharge teaching which was an independent variable. Thirdly, readiness for hospital discharge as the dependent variable was regressed on discharge teaching and social support which were independent variables. At each step, we will control the sociodemographic factors that affect the dependent variable. Regression coefficients for each regression equation were tested.

When the coefficient of the first step is not significant, it represents that the mediating effects analysis is ended. But when the coefficients of the first and second step are significant and the coefficient for the independent variable (discharge teaching) in the third step is not significant, this indicates that it was a full and significant mediating effect. There is another situation, when the coefficients of the first step and second step are significant and the coefficient for the independent variable (discharge teaching) in the third step is less than the result of the second step, this suggests that it was a significant but partial mediating effect.

## 3. Results

### 3.1. Sample Characteristics

A total of 288 patients were recruited for the survey. 33 responses were excluded from analyses owing to incomplete/missing data. Consequently, 255 patients were included in analyses (valid response rate = 88.5%). Samples included 146 males and 109 females, and the average age of the patients was 51.09 ± 13.87. Most samples were married and lived in city, and a total of 182 (71.4%) participants had ophthalmology history. Other demographic characteristics of samples are displayed in Table 1.

### 3.2. The Quality of Discharge Teaching, Social Support, Discharge Readiness, and Their Relationships

The mean total scores among participants in this study for quality of discharge teaching, social support, discharge readiness were 122.97 ± 21.546, 36.32 ± 7.598, and 157.91 ± 26.675, respectively. The statistics of other dimensions are summarized in Table 2.

The quality of discharge teaching was found to be highly related to readiness for discharge (*r* = 0.708, *p* < 0.01). Social support had a positive correlation with the quality of discharge teaching (*r* = 0.370, *p* < 0.01) and readiness for discharge (*r* = 0.461, *p* < 0.01). The correlations among the measured variables are displayed in Table 3.

### 3.3. The Differences among Sample Characteristics, Social Support, and Discharge Readiness

The level of social support differed significantly by samples' marital status (*F* = 33.71, *p* < 0.001), education level (*F* = 24.95, *p* ≤ 0.001), monthly income (*F* = 19.99, *p* ≤ 0.001), living place (*t* = −4.444, *p* ≤ 0.001), and ophthalmology history (*t* = 2.065, *p*=0.040). The status of readiness for discharge differed by sample's gender (*t* = 2.819, *p*=0.005), education level (*F* = 28.57, *p* ≤ 0.001), monthly income (*F* = 10.945, *p* ≤ 0.001), and living place (*t* = -4.541, *p* ≤ 0.001) significantly. The results are displayed in Table 4.

### 3.4. The Effect of Social Support on the Quality of Discharge Teaching and Readiness for Discharge

**Figure 2:**
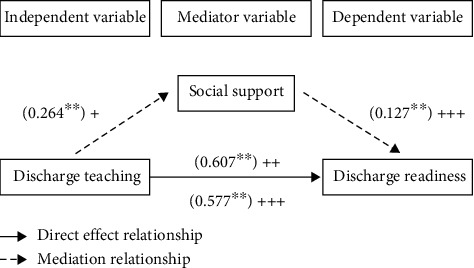
The mediator model of social support. *Note.* The mediator model of social support. ^*∗∗∗*^*p* < 0.05. “+”: the first step represents social support regressed on discharge teaching. “++”: the second step represents discharge readiness regressed on discharge teaching. “+++”: the third step represents discharge readiness regressed on discharge teaching and social support.

The results of regression analysis indicated that social support played a partial mediator role in the relationship between discharge teaching and readiness for discharge (Figure 2). In the first step, regression analysis was performed using discharge teaching as the independent variable and social support as the dependent variable, after controlling the sociodemographic characteristics of samples such as marital status, education level, monthly income, living place, and ophthalmology history; the result was significant (*β* = 0.264, *p* < 0.001). In the second step, readiness for hospital discharge as the dependent variable was regressed on discharge teaching which was independent variable. We also control samples' sociodemographic characteristics (gender, education level, monthly income, and living place) which can affect readiness for hospital discharge. The result means that readiness for hospital discharge was regressed on discharge teaching significantly (*β* = 0.607, *p* < 0.001). In the last step, readiness for hospital discharge as the dependent variable was regressed on discharge teaching and social support which were independent variables after controlling samples' gender, education level, monthly income, and living place. The results indicate that readiness for hospital discharge was significantly regressed on discharge teaching (*β* = 0.577, *p* < 0.001) and social support (*β* = 0.127, *p* < 0.001). Through the analysis of the above results, social support played a mediator role between discharge teaching and readiness for discharge, and the mediation effect ratios were 0.264*∗*0.127/0.607 ≈ 5.5%. The regression coefficients for each regression equation are shown in Table 5.

## 4. Discussion

The study aimed to understand the role of social support in the relationship between discharge teaching and readiness for discharge among fundus disease patients treated with day surgery. It corresponded to the transition theory [40]. In the transition theory, there are four key transition components including nature of transition, transition conditions, nursing therapeutics, and patterns of response. In this research, the nature of transition means the factors that account for the differences in the characteristics of postoperative patients who will discharge to recovery at home or community. Besides, social support from patients was the important transition condition. Then the nursing therapeutics indicates nursing intervention such as discharge teaching to facilitate the transition and help patients grasp new skills and adapt to new roles before patients go home as discharge teaching may influence discharge readiness and the transition to home. In addition, the pattern of response is examined by accessing the readiness for hospital discharge of patients. The objective of this pattern was to improve the understanding of their rehabilitation for patients and to encourage their confidence in self‐care ability and connect with the healthcare community [17]. And the results in this study also showed that participants with stronger social support had better discharge teaching and then had higher readiness for discharge. Social support played a partial mediator role.

The direct relationship between discharge teaching and readiness for discharge was similar to several studies in which discharge teaching had a positive association with readiness for discharge [16], and it can facilitate patients “readiness for discharge to go home” [21]. A high level of discharge teaching can help improve the patient's self-care ability, reduce the patient's sense of uncertainty after discharge, and then increase their perception of discharge readiness [26]. Besides, discharge teaching skills also affect the patient's correct understanding of disease knowledge and self-care skills. The higher the discharge teaching skills are, the more the patient will tend to execute the discharge guidance content correctly after discharge [41]. As mentioned, patients in day wards turn over rapidly in the latter, individual education methods which are used in inpatient wards are probably not suitable in day wards, and education involved a variety of health education activities, including oral and video presentations, written instructions, and demonstrations which can arouse patients' interest in learning disease knowledge and self-care skills after hospital discharge [7]. This suggested that more attention to the individual needs of patients, combined with better understanding of their expectations of discharge teaching, is needed. Medical staff in hospitals should also enrich the styles and content of discharge teaching to further improve the readiness for hospital discharge for patients.

Additionally, discharge teaching not only had direct effect on readiness for discharge but also indirectly influenced it through social support; social support played a mediator role between discharge teaching and readiness for discharge in this study. In this study, social support was measured by social support rating scale, and the scale consists of three dimensions including subjective support, objective support, and support utilization; when objective support was sufficient, patients could use social support more actively, thus promoting the development of coping skills [42]. For patients in hospital, the support system also contains the support from healthcare professionals and family members. During preoperative education, meeting healthcare staff in advance and having the disease information support from healthcare professionals have increased the confidence of patients and their family members for surgery [43] and then it is beneficial for patients' postoperative rehabilitation. If the support from family is strong, family members could increase the education about disease rehabilitation from healthcare professionals of discharge teaching and have a good preparation to fulfill the caregiving role after hospital discharge. A report also indicated that family-centered approach can promote discharge readiness through staff education [44]. If possible, friends or even community health service staff may provide tangible forms of support, which in turn could promote patients to respond to postoperative recovery actively. Therefore, healthcare providers should assess patients' social support early, and their support systems should be involved in the preoperative and postoperative processes.

Moreover, previous studies showed that social support is an important factor that impacts readiness for discharge [15]. When patients do not have enough social support, they are less prepared for discharge from hospital. Lack of social support has been identified as one of the main factors that delays discharge after surgery [45]. Nursing staff should educate family members as caregivers how to support the patient at home to ensure optimal outcomes.

Although discharge teaching can affect readiness for discharge through the mediation of social support, the mediation effect ratios were only 5.5% in this research. It may be that the hospital stay for day surgery patients is relatively short and only the social support during the hospitalization could be evaluated in this study, but the social support after discharged cannot be accurately evaluated. It also suggested that there existed other factors contributing to the relationship between discharge teaching and readiness for discharge. A recent study investigating surgical patients showed that care coordination can predict readiness for discharge and probably plays a mediation role between discharge teaching and readiness for discharge [46]. There may be other mediating factors making a contribution between discharge teaching and readiness for discharge such as patient characteristics and patient self-care ability [15].

### 4.1. Limitations

This study had several limitations. First, the samples were collected from one hospital in Guangzhou, China; therefore, it may not be possible to extend the results to all patients with ocular fundus disease treated with day surgery. Second, we did not explore all mediating factors that may influence the readiness for discharge for patients with ocular fundus disease. More comprehensive and prospective studies concerning readiness for discharge are needed.

## 5. Conclusions

In summary, patients with fundus disease who perceive higher levels of social support and education at discharge may experience a greater readiness for discharge. Social support plays a partial moderating role between discharge education and discharge preparation. Nevertheless, discharge teaching has shown the highest correlation on readiness for discharge but also had indirect influences through the mediating role of social support.

Health professionals especially clinical nurses should provide appropriate discharge guidelines according to the needs of patients and assess the sources of social support among patients before and during treatment in hospital when necessary. Specifically, encouraging patients to proactively utilize the support especially family-based support and health professional-based support, which are important sources of social support to enhance the readiness for discharge of fundus disease patients treated with day surgery, is important.

## Figures and Tables

**Table 1:** General information of samples (*n* = 255).

Variable	*N* (%)/mean (SD)
Age	51.09 (13.87)

*Gender*	
Male	146 (57.3)
Female	109 (42.7)

*Marital status*	
Married	203 (79.6)
Divorced	9 (3.5)
Single	43 (16.9)

*Education level*	
Below primary school	65 (25.5)
Junior high school	60 (23.5)
High school or some college	75 (29.4)
University or above	55 (21.6)

*Monthly income (in RMB)*	
<2000	24 (9.4)
2000–3999	102 (40)
4000–5999	92 (36.1)
≥6000	37 (14.5)

*Living place*	
City	164 (64.3)
Village	91 (35.7)

*Insurance*	
New rural cooperative medical system	35 (13.7)
Resident medical insurance	90 (35.3)
Staff medical insurance	106 (41.6)
No	24 (9.4)

*Ophthalmology history*	
Yes	182 (71.4)
No	73 (28.6)

*Ophthalmic surgery history*	
Yes	155 (60.8)
No	100 (39.2)

**Table 2:** Status on the quality of discharge teaching, readiness for hospital discharge, and social support (*n* = 255).

Dimension	M ± SD	Maximum	Minimum
Teaching contents that patients thought they needed	53.72 ± 9.143	135	2
Teaching contents that patients actually obtained	37.76 ± 8.371	59	11
Teaching skills and effectiveness	85.20 ± 14.489	118	40
Total score of the quality of discharge teaching	122.97 ± 21.546	170	52
Physical conditions	53.28 ± 10.131	70	20
Mastery of disease knowledge	46.74 ± 14.331	76	0
Coping ability after discharge	23.64 ± 4.641	30	8
Available social support	34.25 ± 6.675	40	6
Total score of the readiness for hospital discharge	157.91 ± 26.675	209	66
Subjective support	17.49 ± 3.772	27	10
Objective support	12.40 ± 3.495	22	6
Support utilization	6.43 ± 1.877	11	4
Total score of social support	36.32 ± 7.598	58	22

**Table 3:** Correlation between quality of discharge teaching, readiness for hospital discharge, and social support (*n* = 255).

	RHDS	QDTS	SSRS
Readiness for hospital discharge (RHDS)	1		
Quality of discharge teaching (QDTS)	0.708^*∗*^	1	
Social support (SSRS)	0.461^*∗*^	0.370^*∗*^	1

^*∗*^ *p* < 0.01.

**Table 4:** The differences among sample characteristics, social support, and discharge readiness.

Variable	Social support			Discharge readiness		
	Mean (SD)	*t* or *F*	*P*	Mean (SD)	*t* or *F*	*P*
*Gender*						
Male	37.03 (7.67)	*t* = 1.741	0.083	161.92 (25.58)	*t* = 2.819	0.005^*∗*^
Female	35.37 (7.43)			152.53 (27.27)		

*Marital status*						
Married	38.08 (7.07)	*F* = 33.71	≤0.001^*∗*^	159.23 (26.22)	*F* = 1.391	0.251
Divorced	30.00 (7.25)			148.11 (34.02)		
Single	29.33 (4.98)			153.72 (26.99)		

*Education level*						
Below primary school	30.34 (4.58)	*F* = 24.95	≤0.001^*∗*^	136.48 (26.77)	*F* = 28.57	≤0.001^*∗*^
Junior high school	37.13 (7.60)			158.52 (20.06)		
High school or some college	38.15 (6.18)			165.01 (25.12)		
University or above	40.02 (8.30)			172.89 (18.46)		

*Monthly income (in RMB)*						
<2000	29.92 (5.82)	*F* = 19.99	≤0.001^*∗*^	139.67 (35.96)	*F* = 10.945	≤0.001^*∗*^
2000–3999	33.90 (7.15)			152.30 (25.03)		
4000–5999	39.35 (6.83)			163.36 (22.96)		
≥6000	39.62 (6.77)			171.65 (22.89)		

*Living place*						
City	37.84 (7.40)	*t* = −4.444	≤0.001^*∗*^	163.35 (23.86)	*t* = −4.541	≤0.001^*∗*^
Village	33.58 (7.21)			148.10 (28.74)		

*Insurance*						
New rural cooperative medical system	38.97 (7.09)	*F* = 2.225	0.086	163.37 (24.51)	*F* = 0.573	0.633
Resident medical insurance	36.02 (7.65)			157.40 (27.72)		
Staff medical insurance	36.22 (7.77)			156.76 (25.39)		
No	34.04 (6.70)			156.92 (31.62)		

*Ophthalmology history*						
Yes	35.70 (7.69)	*t* = 2.065	0.040^*∗*^	158.30 (26.72)	*t* = −0.365	0.715
No	37.86 (7.19)			156.95 (26.72)		

*Ophthalmic surgery history*						
Yes	35.65 (7.47)	*t* = 1.777	0.077	160.50 (26.18)	*t* = −1.943	0.053
No	37.37 (7.72)			153.89 (27.06)		

^*∗*^ *p* < 0.05.

**Table 5:** The regression coefficients for each regression equation.

Step	Independent variable	Dependent variable	*β*	*P*	SE	*t*	*F*	*R* ^2^
1	Discharge teaching	Social support	0.264	≤0.001	0.019	5.016	27.175	0.397
2	Discharge teaching	Readiness for discharge	0.607	≤0.001	0.054	13.993	68.676	0.586
3	Social support	Readiness for discharge	0.127	0.009	0.169	2.648	60.149	0.597
	Discharge teaching		0.577	≤0.001	0.055	13.022		

*Note.* In step 1, we controlled the sociodemographic characteristics of samples such as marital status, education level, monthly income, living place, and ophthalmology history which can affect social support; in step 2, we controlled the sociodemographic characteristics of samples such as gender, education level, monthly income, and living place which can affect readiness for hospital discharge; in step 3, we controlled the sociodemographic characteristics as in step 2.

## Data Availability

The data used to support the findings of this study are included within the article. The specific data analysis in a SPSS file are available from the corresponding author upon request.
